# Current Staging Procedures in Urinary Bladder Cancer

**DOI:** 10.3390/diagnostics3030315

**Published:** 2013-06-25

**Authors:** Tobias Maurer, Thomas Horn, Matthias Heck, Jürgen E. Gschwend, Matthias Eiber, Ambros J. Beer

**Affiliations:** 1 Department of Urology, Klinikum rechts der Isar, Technical University Munich, Ismaniger Str. 22, 81675 Munich, Germany; E-Mails: t.horn@lrz.tum.de (T.H.); m.heck@lrz.tum.de (M.H.); juergen.gschwend@lrz.tum.de (J.E.G.); 2 Department of Radiology, Klinikum rechts der Isar, Technical University Munich, Ismaniger Str. 22, 81675 Munich, Germany; E-Mail: matthias.eiber@tum.de; 3 Department of Nuclear Medicine, Klinikum rechts der Isar, Technical University Munich, Ismaniger Str. 22, 81675 Munich, Germany; E-Mail: ambros.beer@tum.de

**Keywords:** urinary bladder cancer, MRI, PET/CT, FDG, choline, actetate

## Abstract

Currently computed tomography (CT) represents the most widely used standard imaging modality in muscle-invasive urinary bladder cancer. Visualization of local tumor or depth of invasion as well as lymph node staging, however, is often impaired. Magnetic resonance imaging (MRI) with diffusion-weighted sequences, determination of apparent diffusion coefficient (ADC) values or utilization of superparamagnetic iron nanoparticles potentially exhibits advantages in the assessment of local tumor or lymph node involvement and therefore might play a role in routine staging of urinary bladder cancer in the future. Likewise, positron emission tomography (PET) with the currently utilized tracers ^18^F-FDG, ^11^C-choline and ^11^C-acetate is investigated in bladder cancer patients—mostly in combination with diagnostic CT. Although promising results could be obtained for these PET/CT examinations in smaller series, their true value cannot be determined at present.

## 1. Introduction

(Urinary) bladder cancer (BCa) is considered one of the most common malignancies worldwide with an incidence of 18.5 per 100,000 males and 5.7 per 100,000 females and approximately 25% of newly diagnosed BCa patients present with aggressive muscle-invasive disease [1]. Standard treatment for muscle-invasive BCa is radical cystectomy (RCX) with pelvic lymph node dissection (PLND) [2]. In patients treated with curative-intended RCX and PLND there is a 40% difference in three year cancer-specific survival (91.4 ± 1.7% *versus* 50.9 ± 3.5%) between those with organ-confined BCa and those with a cancer infiltrating perivesical fatty tissue or metastatic lymph nodes [3]. Especially in cases of locally advanced disease or high-risk disease for development of metastases platinum-based neoadjuvant chemotherapy regimes have been shown to improve patient cure rates while palliative treatment is advocated for metastatic disease [4,5]. The decision for the optimal treatment strategy is mainly based on results of imaging. Therefore accurate pre-treatment staging of these patients is of major importance.

**Figure 1:**
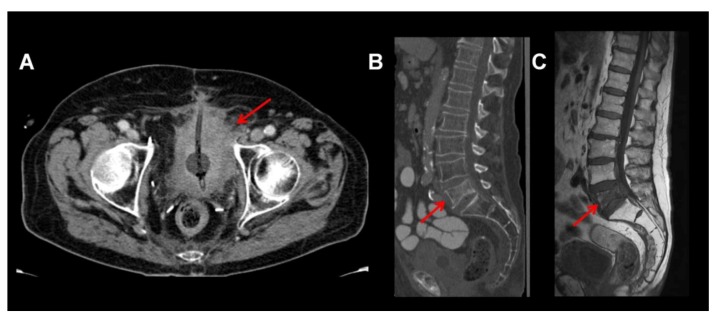
Large bladder tumor visualized by contrast enhanced computed tomography (CT) reaching the acetabulum on the left side and infiltrating the perivesical fatty tissue (**A**). Note there is an additional spinal metastasis in L5 (**B**, sagittal reformatted CT), seen as a destructive lesion in L5 with sintering of the vertebra. This is confirmed by MRI: the lesion is seen hypointense in T1w, confirming infiltration of the bone marrow by tumor tissue (**C**, T1w sagittal).

**Figure 2:**
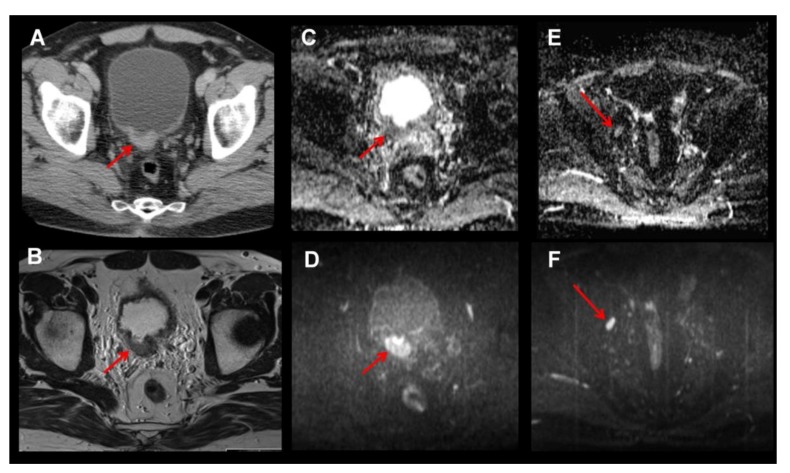
Bladder tumor at dorsal bladder wall visualized by CT as a wall thickening with contrast enhancement (**A**). Of note, soft tissue contrast is superior in MRI compared to CT (**B**, T2w axial). Additional diffusion weighted imaging (DWI; **C**–**F**) improves delineation of the tumor mass as a hyperintense mass in the b-value images (**D**, b800 image) and allows evaluating apparent diffusion coefficient (ADC) values (**C**) as potential marker of tumor cellularity. Moreover, DWI might be helpful for evaluating lymph node metastasis, as metastatic lymph nodes usually also show lower ADC values (**F**, b800 image showing a hyperintense iliacal lymph node on the right; **E**, low ADC value suggests malignancy).

The current standard pre-operative imaging modality represents contrast-enhanced computed tomography (CT) (Figure 1, Figure 2(A)). However, in up to 40% of cases, CT underestimates the disease [3]. It has been reported that CT can only marginally differentiate between tumor stages Ta to T3a and even in cases with macroscopic invasion of perivesical fatty tissue, accuracy rates range from 55–92% [2]. In addition, regenerative and inflammatory postoperative tissue alterations after previous transurethral resection of the BCa further impair exact local T-staging. The sensitivity for detection of lymph node metastases (48–87%) is also disappointing. Despite these facts, the guidelines of the European Association of Urology still recommend CT as the standard preoperative imaging modality, simply due to the lack of proven superior imaging alternatives [2]. 

## 2. MRI

Besides CT, magnetic resonance imaging (MRI) has been evaluated in several studies for staging of muscle invasive bladder cancer (Figure 2(B)). The tumor is usually well depicted on T2-weighted images. For better assessment of perivesical infiltration, the signal from perivesical fat can be suppressed using short tau inversion recovery (STIR) sequences [6]. By using contrast-enhanced imaging with gadolinium-containing contrast, the accuracy of MRI has been reported to be 85% for differentiating non-muscle invasive bladder cancer from muscle-invasive bladder cancer. For distinguishing organ confined disease from non-organ confined disease accuracy of 82% was reported [7]. In their most recent study, Liedberg *et al*. prospectively examined 53 patients prior to radical cystectomy with 3 Tesla MRI and defined bladder filling and compared results of the clinical local tumor staging to histopathological findings. In twenty three patients (49%) final histopathological analysis led to down-staging and in six patients (13%) to up-staging of local tumor. The authors concluded that local tumor staging with MRI is mainly hindered by previous bladder tumor resections and resulting postoperative changes [8]. 

Diffusion-weighted magnetic resonance imaging (DW-MRI) and determination of the apparent diffusion coefficient values (ADC) are modern functional MR-imaging techniques (Figure 2(C–F)). The ADC value describes the ability of water molecules to diffuse in tissue, which is impaired by increased cellular density as is the case in tumors. Kobayashi *et al*. were not only able to detect bladder tumors with a sensitivity of >90% in 104 patients using ADC values, but could also differentiate aggressive high-grade or muscle invasive bladder cancer from low-grade tumors or tumors without muscle infiltration with a sensitivity of 88%, a specificity of 85% and a diagnostic accuracy of 87% [9]. These findings were confirmed in another patient collective (n = 45) examined with MRI and ADC values within the scope of hematuria clarification. However, in this study, benign bladder tumors also exhibited low ADC values rendering differentiation between benign and malignant bladder tumors difficult [10]. MRI was performed before transurethral resection in both studies, this represents an important limitation as the impact of surgical intervention and postoperative changes were not assessed. 

Daneshmand *et al*. performed MRI examinations in 122 patients with BCa after transurethral resection prior to radical cystectomy and observed that MRI was indeed able to improve local tumor and lymph node staging in this patient cohort. They reported sensitivity, specificity and diagnostic accuracy rates of 87.5%, 47.6% and 74%, respectively, for differentiation of patients with organ-confined lymph node negative BCa from patients with non-organ-confined disease or lymph node positive tumors. The sensitivity, specificity and diagnostic accuracy rates of lymph node metastases detection were 40.7%, 91.5% and 80.3%, respectively [11]. In general, for detection of suspicious lymph nodes, especially when they are smaller than 10 mm in diameter, MRI seems to be superior to conventional CT imaging [12,13]. 

DW-MRI can also be utilized for response evaluation in muscle-invasive BCa undergoing neoadjuvant chemoradiation protocols as Yoshida *et al*. were able to demonstrate [14]. In their study, 23 patients with muscle-invasive BCa underwent two cycles of neoadjuvant cisplatin-based chemoradiation followed by radical or partial cystectomy with DW-MRI staging before and after chemoradiation therapy. It could be demonstrated that responders showed significant lower ADC values and the authors concluded that DWI-MRI might therefore be used for prediction of favorable response or even for patient selection with regard to bladder-sparing approaches.

Another interesting functional MRI technique is dynamic contrast enhanced MRI (DCE-MRI), which can be used to evaluate tissue perfusion. Recent data have shown that the results of DCE-MRI correlate with microvessel density, so this technique seems promising for assessment of angiogenesis, maybe also for response assessment [15]. However, its clinical value is not yet determined. 

A promising, rather new development for lymph node evaluation by MRI represents the usage of ultrasmall superparamagnetic iron oxide particles (USPIO) [16,17,18]. For this, an MRI examination is performed before and approximately 24–36 h after injection of USPIO. After uptake of these nanoparticles in macrophages and dendritic cells, the resulting signal loss in lymph nodes can be visualized by direct comparison. If lymph nodes are infiltrated by tumor cells and normal lymphatic cells are displaced no signal loss can be observed in the post-injection MRI and thus the lymph node is likely to harbor metastatic infiltrates. Initially this method showed very promising results with a considerable increase of sensitivity up to 96% (from 76% for MRI without USPIO) and a specificity of 95% [16]. Due to problems with regulatory approval authorities, USPIO is currently available only in a few centers. Just recently collaborative publications by Froehlich and Triantafyllou *et al*. confirmed again these initial excellent results for detection of metastatic lymph nodes in 58 and 75 patients with bladder or prostate cancer. Especially when lymph nodes appear normal-sized on conventional MRI the use of USPIO could be of increased diagnostic value they concluded. However, lymph node metastases under 5 mm can only be detected with uncertainty [17,18]. 

In summary, MRI using DWI has the potential to improve preoperative staging of BCa. Precise and standardized assessment from well-trained radiologists, however, is warranted. Further prospective studies with larger patient cohorts and histopathological correlation are necessary to determine the true value of MRI-based imaging [19]. 

## 3. PET and PET/CT

Positron emission tomography (PET) represents a biological rather than exact anatomical imaging modality, which is nowadays commonly combined with diagnostic CT. Depending on the tracer used for PET imaging metabolism or expression of certain antigens can be visualized and attributed to exact anatomical structures or organs especially when fused to corresponding CT images. For BCa most studied tracers represent ^18^F-FDG, ^11^C-choline (or ^18^F-choline) and ^11^C-acetate. 

### 3.1. ^18^F-FDG

Until now primarily ^18^F-fluorodeoxyglucose (^18^F-FDG; half-life 109 min) has been used as tracer in staging of BCa patients. ^18^F-FDG is taken up into metabolic active cells through glucose transporters, where it is phosphorylated by hexokinase and trapped intracellularly as no further metabolization takes place. However, apart from previous transurethral resections and postoperative reactive changes, especially renal excretion of ^18^F-FDG and subsequent accumulation of the tracer in urine and in the bladder render detection and assessment of local bladder tumors difficult. Some modifications have been introduced in order to bypass these problems, such as flushing the bladder [20,21], application of diuretics or early scanning 2–4 min after tracer injection. Unfortunately, still false-positive findings can be observed [21].

With regard to metastatic disease to lymph nodes four larger studies evaluated patients using ^18^F-FDG-PET/CT before undergoing radical cystectomy and pelvic lymph node dissection [22,23,24,25]. Kibel *et al*. included 43 patients with negative CT as well as bone scans in their study and detected histologically confirmed metastatic lymph node infiltrates in 7 out of 9 patients. PET/CT displayed a sensitivity of 70%, specificity of 94%, a positive predictive value of 78% and a negative predictive value of 91% [22]. Lodde *et al*. reported similar results in 44 preoperative patients. PET/CT also showed a higher sensitivity when compared to CT (57% and 33%, respectively), whereas both modalities had a specificity of 100% in their study [23]. Furthermore, in the subpopulation with metastatic disease, PET/CT could visualize bone metastases confirmed by conventional bone scan.

In a smaller patient cohort with metastatic disease ^18^F-FDG-PET/CT proved to be of value compared to conventional CT or MRI [24,26,27]. However, in the largest ever published study with 51 patients involving 51 patients (including 13 patients with lymph node metastases) by Swinnen *et al*., a significant advantage of ^18^F-FDG-PET/CT over CT alone could not be observed. In this study in contrast to the studies mentioned previously, extensive pelvic lymph node dissection was performed in all patients and the histopathological results were compared at the patient level as well as field-based with the imaging procedures, namely the conventional diagnostic CT alone compared to the complete imaging data set of the ^18^F-FDG-PET/CT examination. In this study, ^18^F-FDG-PET/CT in comparison to CT alone demonstrated a sensitivity of 46% (*vs*. 46%), specificity of 97% (*vs*. 92%) and a diagnostic accuracy of 84% (*vs*. 80%) [25]. 

Although the median sensitivity and specificity rates of ^18^F-FDG-PET/CT were 90% and 100% for local tumor as well as 82% and 89% for lymph node detection in a current meta-analysis of six studies the authors concluded that due to the small number of published studies with only slightly over 300 included patients, no general recommendation on preoperative imaging with ^18^F-FDG-PET/CT can be given [28]. Additionally, in the studies mentioned above, exact histopathological confirmation or comparison between PET/CT and CT examinations were not carried out consequently. Furthermore detection of small or metabolic inactive metastatic lesions seems especially challenging. 

### 3.2. ^11^C-Choline

**Figure 3:**
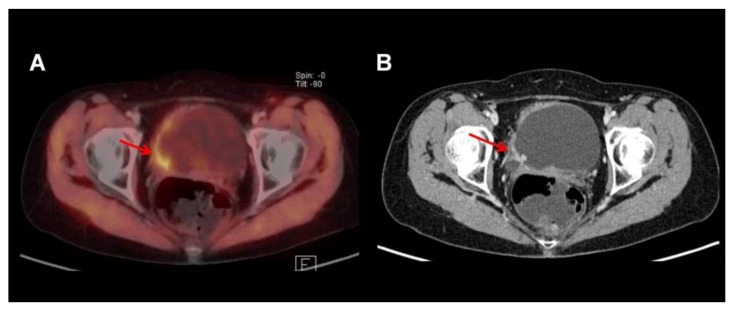
Bladder tumor with tracer uptake in the right posterior-lateral bladder wall as visualized by ^11^Choline-PET/CT. Note that there is excellent delineation of tumor and bladder lumen due to the fact that ^11^C-Choline is usually not excreted by the urine (**A**: fused dataset). However, anatomical detail is superior in the contrast enhanced CT part of the PET/CT (**B**). Note the hypervascularized tumor involving the right ureteral orifice with consecutive dilatation of the right ureter.

^11^C-Choline is another alternative tracer that exhibits a much shorter half-life of 20 min. After cellular uptake, it is integrated into the cell membrane of metabolic active cells. The tracer undergoes predominantly hepatic instead of renal excretion, thus facilitating the evaluation of the bladder and pelvic region (Figure 3). A study of 18 patients by Gofrit *et al*. was able to show promising results in local and systemic tumor staging using ^11^C-Choline-PET/CT [29]. In a study from our institution we performed ^11^C-Choline-PET/CT in 44 patients (12 patients with histological proven metastatic infiltrates in local lymph nodes) prior to radical cystectomy with pelvic lymph node dissection. The results from ^11^C-Choline-PET/CT or diagnostic conventional CT alone (each reviewed by two independent radiologists or nuclear medicine physicians/radiologists) were compared to histopathological findings on a patient level as well as field-based. No significant differences between ^11^C-Choline-PET/CT and diagnostic CT alone regarding local tumor staging, patient-, and field-based analysis were observed. On a patient-level sensitivity, specificity, positive predictive value, negative predictive value and diagnostic accuracy for ^11^C-Choline-PET/CT (in comparison to CT alone) were 58% (*vs*. 75%), 66% (*vs*. 56%), 39% (*vs*. 39%), 81% (*vs*. 86%) and 64% (*vs*. 61%), respectively [30]. Hence, the previously clinical positive reports could not be confirmed in our study—probably by the virtue of the size and metabolic activity of metastatic infiltrates in lymph nodes or presence of inflammatory changes. 

Golan *et al*. published data from a study of 20 patients comparing ^18^F-FDG-PET/CT and ^11^C-Choline-PET/CT noticing that neither tracer performed significantly better than the other [31]. In conclusion, the value of ^11^C-Choline-PET/CT cannot be determined at present, but might be less advantageous than initially hoped for. Its use in the routine diagnostic evaluation of BCa, however, is at the moment not recommended. 

### 3.3. ^11^C-Acetate

Just recently^ 11^C-Acetate, a marker for fatty acid synthase that is overexpressed in several cancers [32,33,34], has been introduced as tracer in BCa staging. Orevi *et al*. compared ^11^C-Acetate-PET/CT to ^11^C-Choline-PET/CT in 14 patients before radical cystectomy and pelvic lymph node dissection, but reported no essential differences between PET imaging with the two tracers. The authors, however, concluded that due to the high negative predictive values of both procedures ^11^C-Acetate-PET/CT or ^11^C-Choline-PET/CT could have implications in the decision-making process either for or against neoadjuvant chemotherapy [32]. Another preliminary study found no advantage of ^11^C-Acetate-PET/CT over MRI or CT [33]. Again, the presence of inflammation after intravesical instillation therapy seems to impair the diagnostic accuracy of ^11^C-Acetate-PET/CT leading to false-positive findings [34]. 

## 4. Conclusions

CT still represents the standard preoperative imaging modality for staging of muscle-invasive urinary BCa although with weaknesses in local tumor and lymph node staging. Modern MRI sequences, such as diffusion-weighted MRI or the utilization of ultrasmall superparamagnetic iron oxide particles (USPIO), show promising results and might eventually play a more important role in staging of urinary BCa. To date, due to the small number of published studies, the clinical value of PET/CT staging with ^18^F-FDG, ^11^C-Choline or ^11^C-Acetate cannot be established, although ^18^F-FDG-PET/CT has been reported in some studies to improve local tumor detection and lymph node staging. Thus, routine PET/CT staging for BCa patients cannot be recommended at present, but should rather be performed in the context of clinical trials [35]. Further developments of more specific tracers for BCa that are not influenced by inflammatory or reactive changes are crucial in order to prevent false-positive findings. Furthermore, it remains to be seen what new diagnostic methods or fusion imaging such as hybrid-PET/MRI can contribute in the future, as it offers the possibility to analyze different metabolic parameters by MRI sequences in combination with functional PET imaging in the context of exact anatomical correlation [36,37].
